# Sentiment and Emotional Analysis of Risk Perception in the Herculaneum Archaeological Park during COVID-19 Pandemic †

**DOI:** 10.3390/s22218138

**Published:** 2022-10-24

**Authors:** Fabio Garzia, Francesco Borghini, Alberto Bruni, Mara Lombardi, Ludovica Minò, Soodamani Ramalingam, Giorgia Tricarico

**Affiliations:** 1 Safety & Security Engineering Group–DICMA, SAPIENZA–University of Rome, 00184 Rome, Italy; 2 Wessex Institute of Technology, Ashurst Lodge, Ashurst, Southampton SO40 7AA, UK; 3 European Academy of Sciences and Arts, A-5020 Salzburg, Austria; 4 Safety & Security Project & Smart Pompeii Project, Pompeii Archaeological Park, Ministry of Culture, 80045 Pompeii, Italy; 5 School of Physics, Engineering and Computer Sciences, University of Hertfordshire, Hatfield AL10 9EU, UK

**Keywords:** cultural sites, risk sentiment analysis, Twitter, opinion mining, OSINT, Herculaneum Archaeological Park, COVID-19 pandemic

## Abstract

This paper proposes a methodology for sentiment analysis with emphasis on the emotional aspects of people visiting the Herculaneum Archaeological Park in Italy during the period of the COVID-19 pandemic. The methodology provides a valuable means of continuous feedback on perceived risk of the site. A semantic analysis on Twitter text messages provided input to the risk management team with which they could respond immediately mitigating any apparent risk and reducing the perceived risk. A two-stage approach was adopted to prune a massively large dataset from Twitter. In the first phase, a social network analysis and visualisation tool NodeXL was used to determine the most recurrent words, which was achieved using polarity. This resulted in a suitable subset. In the second phase, the subset was subjected to sentiment and emotion mapping by survey participants. This led to a hybrid approach of using automation for pruning datasets from social media and using a human approach to sentiment and emotion analysis. Whilst suffering from COVID-19, equally, people suffered due to loneliness from isolation dictated by the World Health Organisation. The work revealed that despite such conditions, people’s sentiments demonstrated a positive effect from the online discussions on the Herculaneum site.

## 1. Introduction

Sentiment analysis and opinion mining have traditionally been used for market exploration to obtain preferential choice on trademarks and goods made in blogs, comments, reviews or tweets. Typically, they are used to extract people’s opinions of certain features on any product. A similar approach may be followed in mitigating the perceived risk by people visiting touristic sites. Typically, security personnel responsible for maintaining the safety of the sites and the security of the visitors would have procedures in place for routine and expected events, incidents or accidents. Even in a monitored environment, it is difficult to perceive any unknown vulnerabilities. When visitors are faced with such risks, their opinions on social media tend to be emotional and acute. It is then necessary to reduce the impact of such emotional opinions and thereby lower the perception of risk at these sites [1,2,3,4,5,6,7,8,9,10,11,12].

Sentiment analysis, also known as opinion mining, is an approach to natural language processing that identifies the emotional tone of a message. It uses data mining, machine learning and AI (artificial intelligence) to mine text for sentiments and subjective information. It involves transforming a set of unstructured text into normalised structured data for analysis. This leads to determining the polarity of data and classifying them into one of many categories such as positive, negative and neutral. 

Italy is known for its priceless cultural heritage and touristic sites that need to be protected, preserved and promoted to attract economic and recreational interest internationally. In a modern world of smart cities where Internet of Things (IoT) and Internet of Services (IoS) form part of an ICT (information and communication technology) platform, opinion mining and sentiment analysis extract opinions of contributors, especially on social media, which are typically classified as negative or positive categories. Thus, sentiment analysis has been an active research area in relation to Italian cultural heritage sites [6,7,8,9,13,14,15,16].

In this paper, we propose a framework of sentiment analysis that considers the emotional associations of visitors with the Herculaneum Archaeological Park (HAP) located in the modern-day Ercolano town, Campania region, Italy. The framework serves as a valuable tool that takes continuous feedback from visitors of cultural heritage sites such as the HAP. Such feedback is captured from social media platforms, namely Twitter [17], to objectively assess the perceived risk and plan mitigation measures to be put in place in time. Such continuous feedback forms a key set of inputs for risk mitigation teams to respond instantly or predict and plan future safety measures based on the perceived risks.

The case study particularly considered an extraordinary period of the COVID pandemic when people were not only suffering from COVID but also from loneliness due to COVID isolation dictated by the World Health Organization (WHO) to prevent any spread of infection. This period signified an emotionally drained period for the general public despite, during which the case study revealed the positive emotional association of people with respect to the HAP. 

### 1.1. Role of Cultural Heritage Sites during COVID-19

The World Health Organisation declared the coronavirus disease-2019 (COVID-19) as a public emergency in January 2020 and as a pandemic in March 2020. As a result, World Heritage sites were closed to visitors. An impact and response tracker published by UNESCO (United Nations Educational, Scientific and Cultural Organization) shows that the ministries of culture in countries such as Argentina, Egypt, Italy and Iran have been emphasizing the development of digital platforms to ensure that knowledge regarding cultural heritage is still being passed on [18]. Thus, they were opened to the public through their digital platforms [19,20]. Such measures helped alleviate problems arising from social isolation and loneliness. Notions of capability, social connections, ontological security and trust—all important elements of wellbeing—are widely shared values. Moreover, cultural heritage sites can bring peace and reconciliation by bringing communities together on a digital platform. 

Since the pandemic, there has been growing research that has analysed the importance of cultural heritage sites and their contribution to the wellbeing of people who did not have the opportunity of visiting these sites. The work in [21] considers in general the benefits of cultural heritage sites in terms of collective wellbeing by connecting people online with their area, fostering a sense of place and belonging. They cite studies that have established a relationship between wellbeing and cultural heritage, the happiness associated with archaeological excavations, the therapeutic benefits of heritage conservation and their stress-relieving aesthetic experience.

The UK Heritage Alliance in its report [22] stresses the importance of cultural heritage including sites in rebuilding society after the impact of COVID-19. Through its case studies, it brings out the benefits of physical and positive impact on mental wellbeing. This study is one of the few to consider the perception of risk, security and safety of heritage sites, contents and visitors during the pandemic, emphasising on the need for risk readiness during health disasters, including man-made and natural hazards [23]. These primarily relate to impact of health crises on the cultural heritages owing to the stopping of maintenance and renovations at these sites. In our previous works, we considered the physical security aspects of cultural heritage sites by means of integrated technological systems [24,25,26,27] as well as the emotional perception of risk from a visitor’s perspective of cultural heritage sites in relation to cultural heritage sites, much before the COVID-19 pandemic period [1,6,7,8,9], and related to different contexts during the COVID-19 pandemic period [10,11,12]. In our proposed work here, we did not relate our research to physical security. Instead, our aim was to find if people’s emotions were positively impacted through online discussions and engagements. Hence, this formed one of the key research questions for this study.

### 1.2. Social Media Role in the Semantic Analysis of Cultural Heritage Sites

Social media platforms such as Facebook, Twitter and WhatsApp influence tourism due to the availability of real-time data providing insights into the fine details of visited sites through users’ digital traces. One could share their experience of what places to see, where to stay, how to move around and the experience they have had during the visits of the sites. This influences the further growth of tourism. Thus, web platforms are media for users to generate content and initiate discussions. For site organisers, the platforms provide insights for more effective planning, management and promotional activities.

Social media platforms have played a key role in keeping up the spirits of people during the COVID-19 pandemic. Researchers have used the data from such platforms to connect the emotions of people with cultural heritage sites. For example, the work in [19] carried out sentiment analysis on data captured from two Instagram hashtags that were widely promoted by UNESCO. Twitter as a social media platform provided a hub for community interaction during COVID-19. The pandemic led to uncertainties and changes that governments and health authorities had to ramp up risk communication measures. Twitter was particularly effective in visually communicating health and risk messages [28]. Similarly, the work in [29] is a case study on Facebook posts in Greece during its great recession period in 2007 when the archaeological discovery in Amphipolis of an apparent tomb of Alexander the Great turned it to once again a cultural cradle of the world. This was made possible by journalism on social media. A thematic qualitative analysis was carried out on all posts and comments that were pre-processed. A qualitative analysis was carried out on how newspapers used social platforms in propagating information related to cultural heritage sites, in particular the discovery of the tomb of Alexander the Great in 2007. The posts of journalists from selective newspapers on social media captured how a depressing country in debt during recession had, in a single day, redeemed its glory from the excavation, boosting people’s morale.

We considered the involvement of the main authors of our proposed paper in research activities related to cultural heritage sites from a risk perception perspective [6,7,8,9]. In [6,7,8,9] the authors carried out sentiment and emotional analysis of visitors’ experiences in different UNESCO cultural heritage sites before the COVID-19 pandemic such as the Papal Basilica and Sacred Convent of Saint Francis in Assisi [6], Pompeii Archaeological Park [7], the Royal Palace of Caserta [8] and the Herculaneum Archaeological Park [9], using the same methodology of the present work, which has been refined and improved over time.

The authors of [13,14] carried out sentiment analysis of visitors’ experiences inside cultural heritage sites with a view to extend services offered similar to the Trip Advisor app. Their proposed work is a natural extension of such a study that applies to the pandemic period. The study in [15] considered a geostatic and textual analysis of cultural heritage sites by using multimedia content and gamification techniques. In [16], the work considered the territorial well-being (quality of life) from demographic data of two Italian provinces and a sentiment analysis of online content related to the sites in these territories. The authors attributed this association to the sharing of digital content and re-creating experiences and perceptions of heritages particularly because they related to their own cultural practices as well as with a view to the function of living heritage sites.

### 1.3. Related Work on Semantic and Sentiment Analyses of Cultural Heritage Sites

The study and analysis of human behaviour using an automated approach is known as sentiment analysis or opinion mining [30]. Social media platforms generate a huge amount of data, and the mining of these data enables the discovery of hidden information. It typically associates opinions into pre-set categories such as positive, neutral or negative emotions. The process of determining an average inclination to any opinion or sentiment enables both organisations and individuals to obtain the right opinion (reviews in retail or tourism sectors) about ongoing trends or unfamiliar aspects. Often, semantic and sentiment analysis are used as synonyms. Semantic analysis is a technique to create a structure around unstructured data such as blogs, posts, social network chatter, tweets, etc., that are not necessarily related. It performs clustering based on similarity, enabling what people think of a particular product, service or experience to be examined. 

A broad set of techniques for sentiment analysis including sentiment polarity, probabilistic, machine learning and artificial intelligence is detailed in [14]. In [13,14], a probabilistic approach to evaluating sentiments called Mixed Graph of Terms, derived from the Latent Dirichlet Allocation (LDA) approach of selective topics, was used for sentiment classification. The theoretical framework for LDA and how relationships between words are learnt is provided in [13]. The work considered Pompeii as their case study with data from both Twitter and Facebook during June 2018. It captured tourists’ experiences within the site and compared before, during and after their visits. The work demonstrated the potential to improve tourism and extend the applications. The LDA technique has an advantage of the graph providing weighted word pairs that are proven to be discriminative for sentiment classification. For these reasons, this is the approach that inspired our work. A key feedback based on sentiment analysis from tweets and Facebook posts brought about changes in the way tickets were sold, made archaeological site information available that made visitors be prepared for rainy days and re-organised the artifacts, significantly improving the feedback. Similarly, in [15], the work considered the promotion of the Italian cultural heritage sites of the Salento territory using a mobile app that was launched at the folk music festival called La notte della Taranta in 2015. A combination of gamification, augmented reality and textual sentiment analysis based on Latent Semantic Analysis was carried out to determine choice of interests.

The work in [19] used the LDA approach for topic modelling followed by a sentiment analysis to understand the cognitive and affective dynamics that emerged from sharing cultural heritage content during COVID-19. Other researchers have set up data collection from Twitter at a very early stage even one week before the first-reported case in Wuhan, 10 May 2020 [20]. Data analysis was carried out on a weekly basis using regression analysis to remove any anomalies followed by sentiment analysis. The key findings from this study on China’s social media WeChat platform were that it was very informative, particularly at the early stages of the pandemic, with high uncertainties. At the same time, an excessive use of social media information on reported cases and their adverse effects affected the mental wellbeing of users. A break from social media was in fact recommended from the study.

Thus, we see recent work associate cultural heritage sites with positive emotions as they relate to their own cultural practices, as well as keep the function of heritage sites alive [19]. In the present work, we adopted a mixed approach where the collecting and first classification of data was made by means of a social network analysis tool (NodeXL) while the detailed classification and analysis of data was made by means of a suitable survey, as outlined in our previous works [6,7,8,9] to avoid the typical errors of automatic tools. However, the authors in [19] noted that during the COVID-19 period, hashtag sampling had dramatically reduced, and, for that reason, this period was excluded in their study. This is one of the key differences from our work, in that we focussed specifically on this very period of the pandemic despite the number of posts, in our case from Twitter, being relatively small. It is also interesting to note that the Wuhan study through the survey on WeChat [20] just before the pandemic had started their work with 558 participants from all seven Wuhan districts. In the early stages of the outbreak, 238 participants had dropped out and treated as missing data for the analysis. Hence, only 320 participants had participated in the survey. Our work here had a comparable sample data through which the findings are made. We also considered tweets in both the English and Italian languages, whereas other work such as [28] compared geographically different places, for example, one being more advanced than the other. Otherwise, the technical approach was quite similar.

Our work addressed the following research questions:

RQ1: Despite the COVID-19 pandemic isolation and loneliness, did discussions on social media create a positive impact on people? How did these compare to the pre-COVID-19 situation as reported in our previous studies?

RQ2: How was the perception of risk related to the well-being in this kind of cultural heritage site during the COVID-19 pandemic?

RQ3: Given that the sample data was small, could we relate our findings to our previous work during the pre-COVID-19 time period to show conclusive evidence to our findings?

The rest of the paper is organised as follows. In Section 2, a description of the Herculaneum Archaeological Park as a UNESCO world heritage site is described as well as the purpose of choosing this site as a case study for sentiment analysis. Section 3 outlines the methodology for sentiment opinion. In Section 4, we provide the results of the sentiment analysis during COVID-19, and we compare them with the results obtained before COVID-19 [9]. In Section 5, we illustrate the limitations of this study and further work.

## 2. Herculaneum Archaeological Park

The Herculaneum Archaeological Park (HAP) is famous for its terrible past with the catastrophic Vesuvius volcanic eruption in 79 A.D. that interred the entire city and all its inhabitants as well as large part of Pompeii. It was only in the 18th century that investigations took place. Worldwide, Herculaneum along with Pompeii became notorious for the disaster from the volcanic eruption as well as being subjected to the looting of priceless possessions from the past centuries. Despites all these disasters, the cities bearing the archaeological sites with historic and artistic values have always remained intriguing and attractive to thousands of visitors each year. In 1977, UNESCO confirmed Herculaneum, Pompeii and their neighbouring regions as a world heritage site.

Herculaneum marvels with its mosaics, painting and houses juxtaposing with its extreme environment and weather conditions. The historic city’s preservation is ever more demanding and necessary due to the weathering effects of material and structural weakening, corrosion by rainwater and the large inflow of tourists into the city, to name a few. 

**Figure 1:**
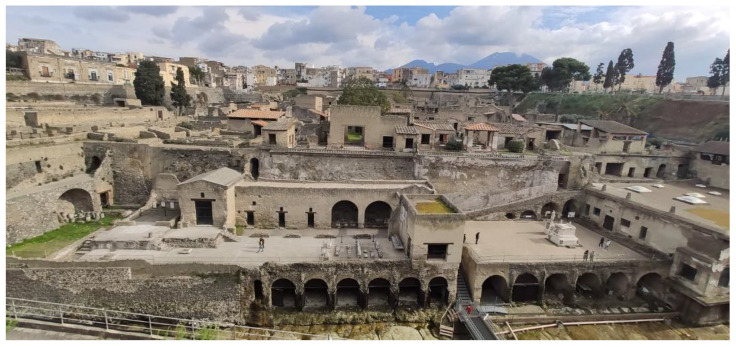
View of Herculaneum Archaeological Park with actual city and Vesuvius volcano in the back [31].

**Figure 2:**
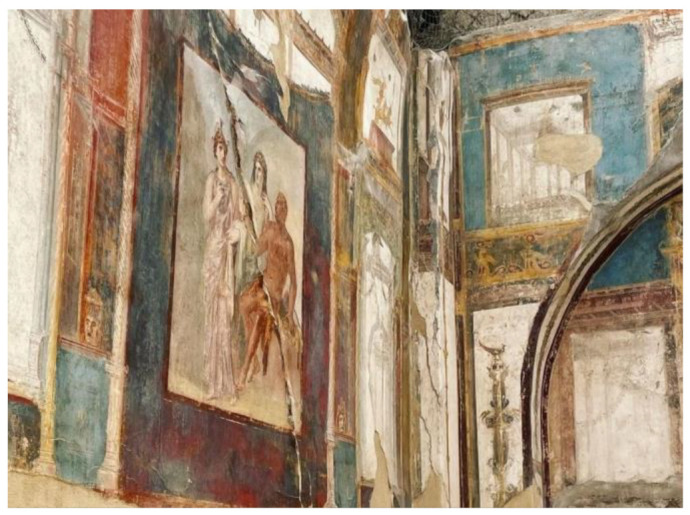
View of the interior of a domus of Herculaneum Archaeological Park [31].

**Figure 3:**
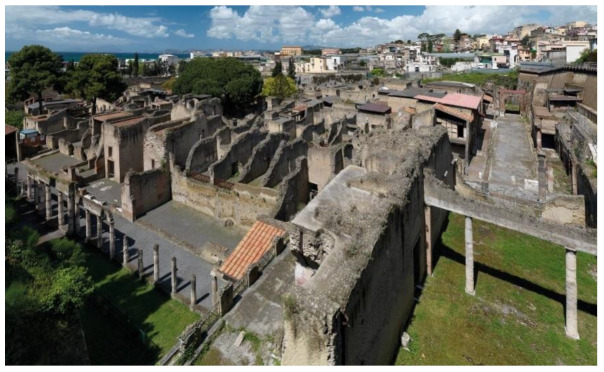
View of Herculaneum Archaeological Park with actual city and gulf of Naples in the back [31].

**Figure 4:**
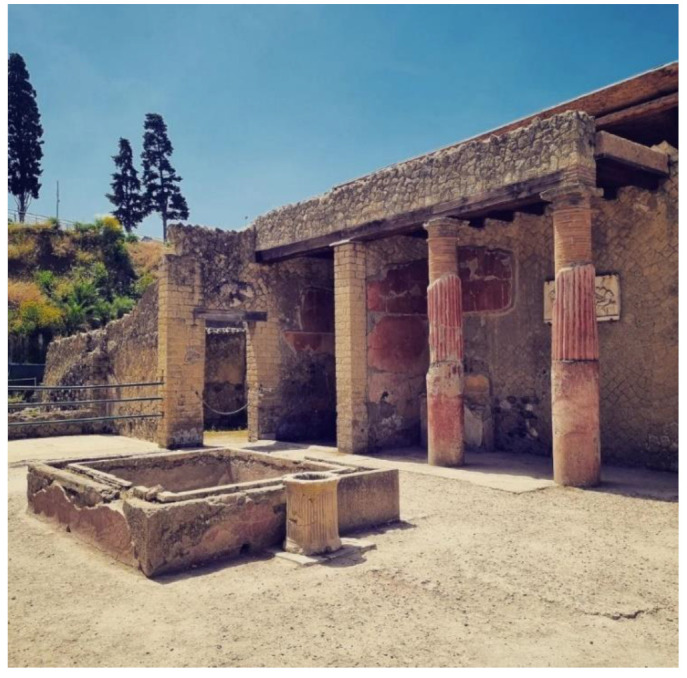
View of the domus of relief of Telephus [31].

**Figure 5:**
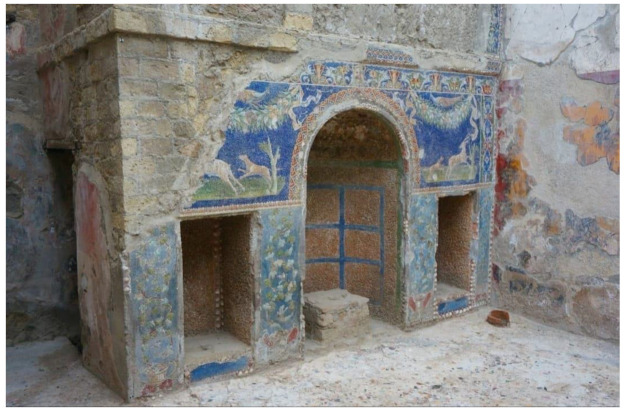
View of the interior of the domus of Neptune and Amphitrite [31].

Some pictures regarding the Herculaneum Archaeological Park are shown in Figure 1, Figure 2, Figure 3, Figure 4 and Figure 5 [31].

Herculaneum’s original city expands over 20 hectares, most of which is buried under the new town built around the archaeological sites across 4 hectares partitioned into 12 zones named ‘insulae’ and includes even the ancient walls and the close Villa of the Papyri. In the site are visible numerous houses (named domus), paintings, mosaics and other artefacts. Such an exceptionally unique site unavoidably provokes emotions in visitors influencing their perception of the risk in the studied location.

- Complex cultural sites such as Herculaneum require special effort to:
- Ensure security and safety;
- Preserve and protect cultural heritage;
- Provide disabled visitor accessibility;
- Provide worker accessibility for site management.

The above objectives can be met by using an integrated systems approach [24,25], which, using the Internet of Things (IoT) and Internet of Everything (IoE), can integrate people, things (mobile terminals, wearable devices, smart sensors, devices, etc.), data, information, knowledge and activities [26,27]. For a site such as Herculaneum, this calls for a multidisciplinary model for security and safety management [32] along with a risk assessment [33,34,35,36,37,38]. For that reason, a suitable project called “Safety & Security for Herculaneum Archaeological Park” was started.

## 3. Semantic Analysis Methodology

**Figure 6:**
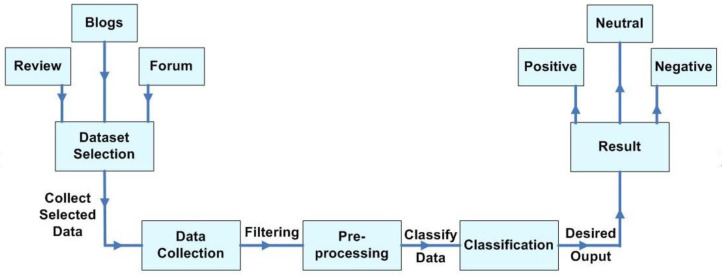
General opinion-mining steps.

Semantic analysis is slightly different from topic-based text classification (sentiment analysis) where classification is carried out on the basis of predefined topics and the topic-related words are important. Instead, opinion words such as positive, negative or neutral are given importance in semantic analysis. In this work, we considered a sentence-level classification as expressing a positive or negative opinion. We discussed key tools followed by the methodology used in this work:

1. Twitter: Twitter [17] was selected as the data source since it is one of the most popular microblogging and social networking sites used by people today. Its usage spikes during prominent events. For instance, it has seen dramatic growth in 2020, possibly due to the COVID-19 pandemic. Twitter revolves around the principle of followers. This tends to influence the opinion of others. From a machine learning perspective, Twitter messages are convenient to use as they are restricted to 280 characters/tweet and lend themselves to a simple, structured way of analyzing the sentence effectively. In this work, tweets in English and Italian were considered that related to the Herculaneum Archaeological Park. Our aim was to assess the emotions of people and estimate their risk perception during the COVID-19 pandemic.
2. NodeXL: NodeXL is a social network analysis tool with a free plug-in for Excel [39]. It facilitates the task of acquiring the data from the social network, storing, analysing and visualising the data, and producing report generation with insights into connected structures. NodeXL uses data-mining techniques, and the advanced NodeXL Pro version includes libraries for machine learning and graph analysis. A general flow diagram in Figure 6 shows these stages. The keywords utilized as seek word are shown in Table 1, during the considered data collection phases shown in Table 2. Table 3 and Table 4 provide typical categories of words related to visitors’ experiences of HAP and the frequencies of occurrences of these words.

The sentiment feature in NodeXL allows users to determine what words matter for a specific purpose. The text analysis features in NodeXL allows the user to edit the lists of words present in the collected tweets that are to be skipped or categorized as List #1, #2, or #3 (that, in our case, were positive, negative and COVID-19, respectively). NodeXL comes with a default collection of American English language “Positive”, “Negative” and “Angry/Violent” terms. It is possible to modify, replace or rename these lists. It is also possible to use any language to populate these lists.

In order to use NodeXL, Twitter data importers require a Twitter account, and, for this reason, a proper account was created for the purpose of this work. Before the first data import, it is necessary to authorize NodeXL by entering a token number, which is automatically sent during the authorization process. Moreover, Twitter’s public free API has many limits. Twitter controls its API and throttles it based on unknown parameters. NodeXL is therefore affected by these limits. Further, all the privacy issues are therefore dependent on the privacy policy of NodeXL, since it represents the tool used to collect tweets. We took measures to protect personal account details during the data collection and processing, focusing only on the text of tweets, even if their accounts were public and accessible on Twitter.

**Figure 7:**
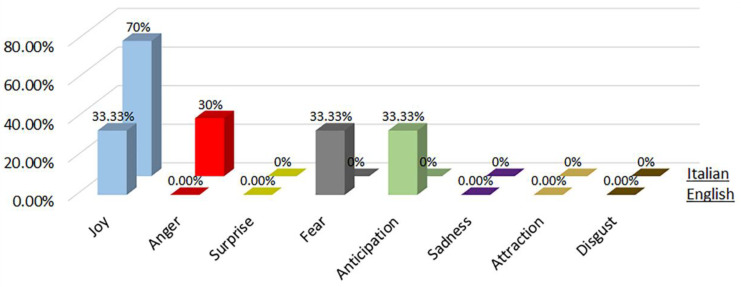
Percentage distribution of every primary emotion for English and Italian words during phase 1 of COVID-19 pandemic.

**Figure 8:**
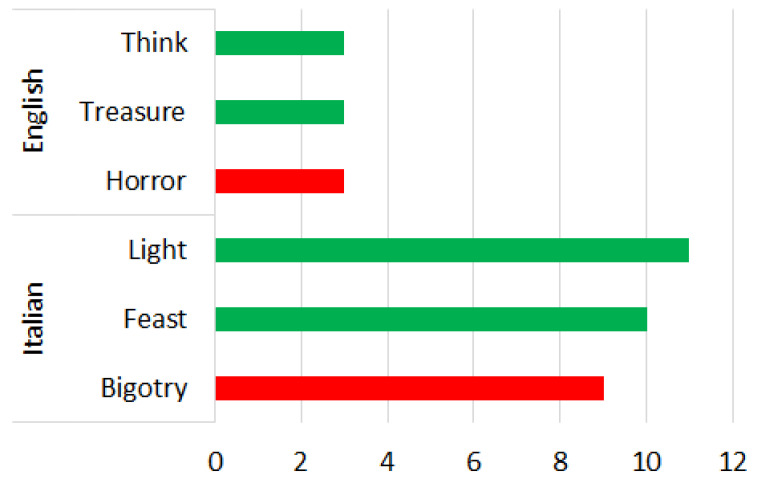
Rating of the most-cited words in English and Italian during phase 1 of COVID-19 pandemic (green is positive, red is negative).

**Figure 9:**
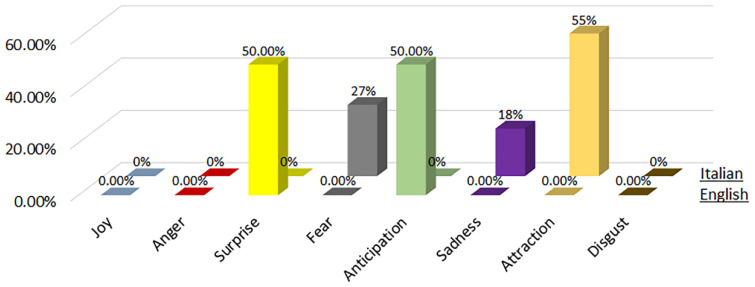
Percentage distribution of every primary emotion for English and Italian words during phase 2 of COVID-19 pandemic.

**Figure 10:**
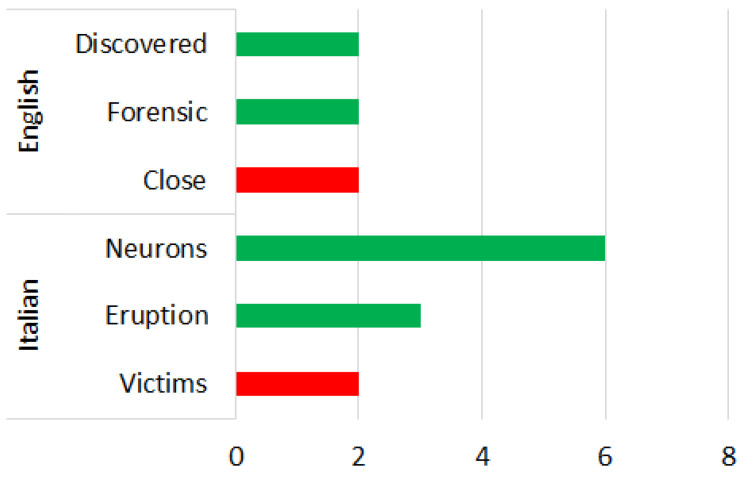
Rating of the most-cited words in English and Italian during phase 2 of COVID-19 pandemic (green is positive, red is negative).

**Figure 11:**
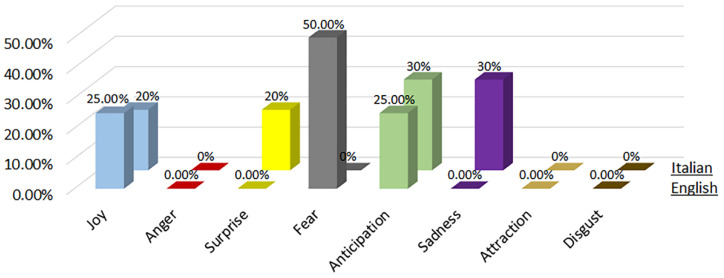
Percentage distribution of every primary emotion for English and Italian words during phase 3 of COVID-19 pandemic.

**Figure 12:**
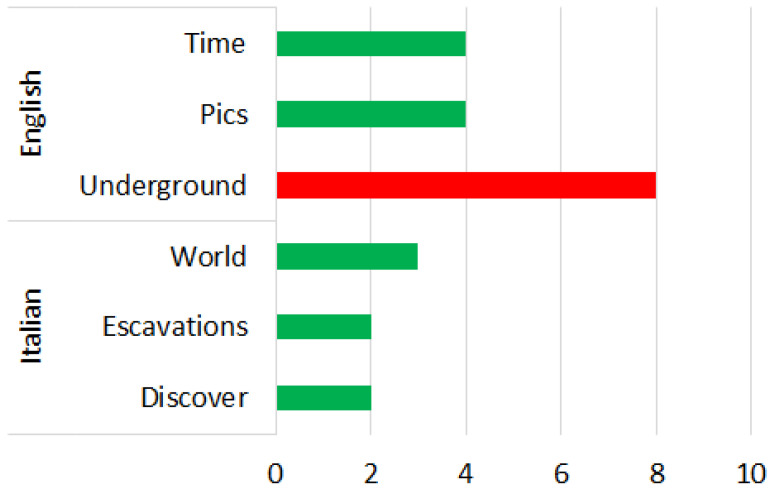
Rating of the most-cited words in English and Italian during phase 3 of COVID-19 pandemic (green is positive, red is negative).

3. OSINT Tool: We considered the Open-Source Intelligence (OSINT) software, which relates to gathering data from open sources, to produce actionable intelligence that is typically used by analysists using non-sensitive intelligence. As we used Twitter as our OSINT source, we chose a set of keywords from tweets that related to perceived risk by people. These keywords correlated to an emotional experience of an individual for a specific site. The work was tested on a Twitter site related to the Herculaneum Archaeological Park (HAP) in Italy during the COVID-19 pandemic.
4. Procedure:
  There were two main steps involved in this analysis:
  1. First, a group of positive and negative words were derived using NodeXL from Twitter words. The purpose of this step was to consider only the most important and recurrent words applicable for our purpose.
  2. Next, a subset consisting of the top three popular words from the above set were subjected to participant survey and they were assigned a polarity (positive, negative) as well as an emotion classification (See Figure 7, Figure 8, Figure 9, Figure 10, Figure 11 and Figure 12. In particular, Figure 8, Figure 10 and Figure 12 for word lists and Figure 7, Figure 9 and Figure 11 for emotions classification).

**Figure 13:**
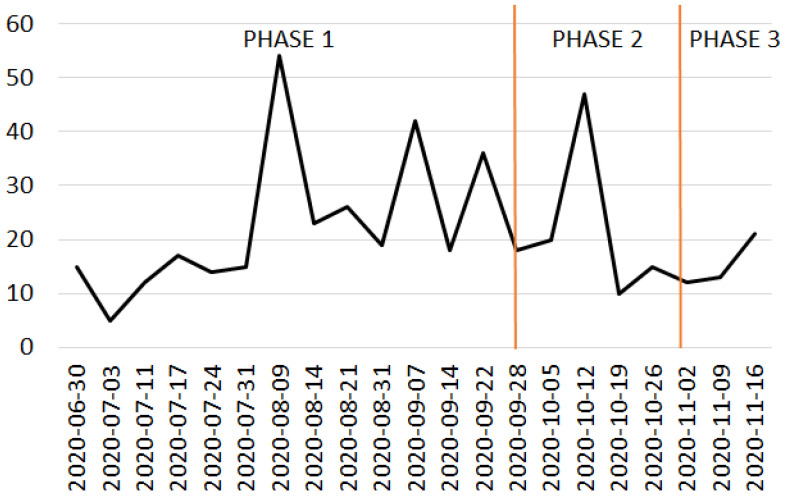
Studied timeline of tweets during COVID-19 pandemic.

Step 1 was repeated for each of the individual phases indicated in Figure 13.

Step 2 was carried out at the end of all the three phases using the aggregated words. Since each phase produced a top-three results, the aggregated list had nine words.

It is to be noted that although both English and Italian dictionaries can be accessed easily, for the specific example site, we produced a novel lexicon of statements. Historically, Herculaneum has been associated with devastation by the Vesuvius volcano depicted by a sense of fear amongst people. Hence, there is sufficient knowledge of words contributing to the negative list that it is populated with. Similarly, any positive mood should also be captured under the positive list. For this purpose, a suitable survey was designed, and inputs collected from 41 evenly distributed male and female participants. They were aged between 17 and 70. This survey was later analysed, which gave an insight into how people map words with emotions. Such a mapping was obtained for every word considered in this work.

## 4. Results of Sentiment and Emotional Analysis from Twitter

The survey started during the COVID-19 pandemic from 30 June to 16 November 2020. During the above considered period, three distinct phases were identified and analysed. The three phases are shown in Table 3. The managing of the data involved generating a certain quantity of information associated to the studied collecting period. They are illustrated in Table 3 and Table 4. 

In order not to overburden the questionnaire to be administered to the sample of people considered, only the three words, positive and/or negative, most mentioned in each phase were considered, both for the English language and for the Italian language.

A group called ‘non-categorized words’ was also included. It considered the words which did not have positive or negative valence. Therefore, we did not consider the words from this group.

As it is possible to see from Table 3 and Table 4, the words in sentiment list#3 related to COVID-19 were empty both for English and Italian. This let us suppose that the HAP had a positive catalysing effect, making visitors forget the health problems of the period under consideration, as is demonstrated in the following by the results obtained.

Figure 13 illustrates the distribution of the number of tweets across the studied timeline of the three phases during the COVID-19 pandemic. We can see a growing/oscillating trend in phase 1 of the curve, which can be attributed to the summer season, where reduced restrictions were present. In phase 2 of the curve there is a peak. This was due to the exceptional discovery of human neurons 2000 years old in that period in the HAP. This represented a unique case in the history and hence there was an attraction to the site. As expected, we can see a descending behaviour because of the starting of new closures due to COVID-19. In phase 3 of the curve there is a slight increase, even if there was a reduction in the mean value due to the restrictions of the considered period.

In our work, tweets were collected each week since NodeXL, in the basic version, has a limit of 2000 tweets of the entire tweet-volume on Twitter at a given moment and can collect tweets from the past 8–9 days. Due to the nature of the considered period and the consequent reduced number of visitors, our collection was far less than this threshold, as seen in Figure 13 and in Table 3 and Table 4. Despite these small numbers, we assumed that the samples were representative and suitable to answer our research questions.

Each of these phases were analysed in detail as follows, showing the results related to the distribution of each primary emotion and sentiment analysis with English and Italian words obtained from the survey:

### 4.1. Phase 1—English Words

During the phase 1 sentiment analysis with English words, we considered the distribution of each primary emotion and the rating of the most-cited words as shown in Figure 7 and Figure 8, respectively. From Figure 7, we can see that the predominant positive emotions arising from the experience of visitors were ‘Joy’ (33.3%) and ‘Anticipation’ (33.33%). The unique negative emotion was represented by ‘Fear’ (33.33%). The emotions of ‘Anger’, ‘Surprise’, ’Sadness’, ‘Attraction and ‘Disgust’ were not present.

### 4.2. Phase 1—Italian Words

During the phase 1 sentiment analysis with Italian words, we considered the distribution of each primary emotion and the rating of the most-cited words as shown in Figure 7 and Figure 8, respectively. From Figure 7, we can see that the predominant positive emotion arising from the experience of visitors was ‘Joy’ (70%). The unique negative emotion was represented by ‘Anger’ (30%). The emotions of ‘Surprise’, ‘Fear’, ‘Anticipation’, ’Sadness’, ‘Attraction’ and ‘Disgust’ were not present.

### 4.3. Phase 2—English Words

During the phase 2 sentiment analysis with English words, we considered the distribution of each primary emotion and the rating of the most-cited words as shown in Figure 9 and Figure 10, respectively. The predominant positive emotions arising from the experience of visitors were ‘Surprise’ (50%) and ‘Anticipation’ (50%). The emotions of ‘Joy’, ‘Anger’, ‘Fear’, ’Sadness’, ‘Attraction’ and ‘Disgust’ were not present.

### 4.4. Phase 2—Italian Words

During the phase 2 sentiment analysis with Italian words, we considered the distribution of each primary emotion and the rating of the most-cited words as shown in Figure 9 and Figure 10, respectively. The predominant positive emotion arising from the experience of such visitors were ‘Attraction’ (55%). Negative emotions were represented by ‘Fear’ (27%) and ‘Sadness’ (18%). The emotions of ‘Joy’, ‘Anger’, ‘Surprise’, ‘Anticipation’, and ‘Disgust’ were not present.

### 4.5. Phase 3—English Words

During the phase 3 sentiment analysis with English words, we considered the distribution of each primary emotion and the rating of the most-cited words as shown in Figure 11 and Figure 12, respectively. The predominant positive emotion arising from the experience of such visitors were ‘Joy’ (25%) and ‘Anticipation’ (25%). The unique negative emotion was represented by ‘Fear’ (50%). The emotions of ‘Anger’, ‘Surprise’, ’Sadness’, ‘Attraction’ and ‘Disgust’ were not present.

### 4.6. Phase 3—Italian Words

During the phase 3 sentiment analysis with Italian words, we considered the distribution of each primary emotion and the rating of the most-cited words as shown in Figure 11 and Figure 12, respectively. The predominant positive emotions arising from the experience of such visitors were ‘Anticipation’ (30%), followed by ‘Joy’ (20%) and ‘Surprise’ (20%). The unique negative emotion was represented by ‘Sadness’ (30%). The emotions of ‘Anger’, ‘Fear’, ‘Attraction’ and ‘Disgust’ were not present.

## 5. Discussion of the Analysis of English and Italian Texts during COVID-19 Period

**Figure 14:**
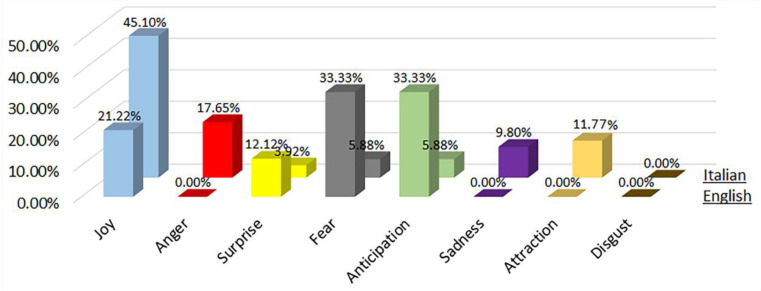
Percentage distribution of every primary emotion for English and Italian words of the 3 phases during COVID-19 pandemic.

The distribution of each primary emotion for English and Italian words of the three phases are shown in Figure 14, from which we can see the prevalence of positive emotions was evident in both languages. The predominantly negative emotional experiences were very limited and associated with words that related to the history and eruption of the volcano Vesuvius that buried the ancient city of Herculaneum than to the place itself. An example is represented by the words victims in Italian and horror in English. References to the coronavirus were practically non-existent.

We can therefore conclude that the perception of risk was low, as all the emotions considered were predominantly positive. The negative emotions related to past adverse events, such as the eruption and its victims. The English term that was most used was ‘time’ (as shown in Figure 12), while the Italian term was ‘light’ (as shown in Figure 8). This underlines, from the psychological point of view, the satisfaction, and the wonder that visitors felt when they went to visit the discovery of a city buried for centuries.

**Figure 15:**
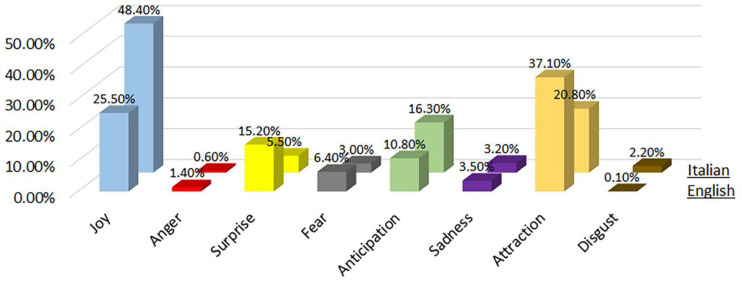
Percentage distribution of every primary emotion for English and Italian words before COVID-19 pandemic [9].

In Figure 15, the distribution of each primary emotion both for English and Italian words of the results obtained before COVID-19 pandemic is shown [9].

The comparison between the results obtained before [9] and during the COVID-19 pandemic showed the general prevalence of positive over negative emotions. This allowed us to conclude that the place prevailed over the circumstances, demonstrating that the Herculaneum Archaeological Park has its own power and is able to give confidence and serenity, despite the surrounding conditions being adverse. This was also confirmed by the absence of words in list #3 of COVID-19-related words of Table 3 and Table 4. Further, the general prevalence of positive emotions in both the cases also allowed us to positively answer the RQ3 of this work since, even if the sample data was small, the findings of our previous work during the pre-COVID-19 period fit to the results obtained in this paper.

We can now address the research questions posed in the beginning of our paper.

RQ1: The period during COVID-19 has been stressful for people across the globe. Cultural heritage sites were closed due to health risks [18,21,22,23]. Hence, these sites moved to digital platforms and found solace by providing virtual experiences and discussions on matters close to communities. New discoveries and excavations also brought the spirits of communities up and changed the perspective of nations through such excavations [29]. Social media did seem to raise the spirits of people even under such trying circumstances. This was reflected by our analysis, re-iterating the fact that it did have a positive impact on communities and a similar surge in positive mood appeared during the second phase after the discovery of human remains. Even though the overall tweets during the pandemic had a dramatic reduction, the distribution of emotions was the same. With reference to the sample data, the total number of tweets collected during the pre-COVID 19 analysis [9] (from 9 June 2019 to 21 July 2019) was 860, with a daily collected tweets mean rate equal to about 20, while the total number of tweets collected during the period of this work (from 30 June 2020 to 16 November 2020) was 452 with a daily collected tweets mean rate equal to about 3.

**Figure 16:**
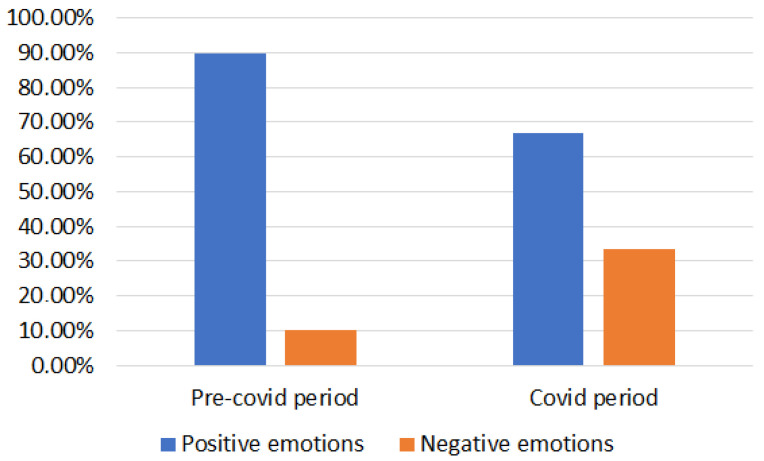
Percentage distribution of primary positive and negative emotions both for English and Italian words before and during COVID-19 pandemic.

RQ2: The only, but vital, risk during the COVID-19 pandemic was health due to the spread of the virus. Since the cultural heritage sites were closed or distance measures adopted, health risks did not prevail much in the discussions on Twitter in relation to the HAP. At the same time, people were in isolation, and we know from reports across the globe the impact that had on people’s well-being [19,20]. What our study shows is that the HAP produced a positive impact under these conditions. With reference to the sampled data, the percentage distribution of primary emotions was equal to about 90% for positive emotions and about 10% for negative emotions during the pre-COVID 19 analysis [9] while it was equal to about 67% for positive emotions and about 33% for negative emotions in the present work. These results are summarized in Figure 16.

RQ3: Our past work [9] provided a report on the pre-COVID-19 period. In comparison to the present day, we can conclude that, pre-COVID-19, Twitter posts were dominated with total positive emotions with a high density of ‘Joy’ (36.95%), ‘Attraction’ (28.95%), ‘Anticipation’ (13.55%) and ‘Surprise’ (10.35%). However, during the pandemic, there was a definite, but small, peak in negative emotions related to ‘Fear’ (19.61%), ‘Anger’ (8.82%) and ‘Sadness’ (4.90%). Hence, even though our qualitative survey and word association with emotions may say the contrary, there was clear indication of some sense of anxiety and fear related to health risks. Nevertheless, the discussions surrounding the HAP and the new discoveries had a positive impact as exhibited by the words related to ‘Joy’ (33.16%), ‘Anticipation’ (19.61%), ‘Surprise’ (8.02%) and ‘Attraction’ (5.88%).

## 6. Limitations of This Study and Further Work

The limitations of this study were represented by the fact we only used Twitter, without considering other social networks where other useful information could be collected. Another limitation was derived by using text only, even if it represented the main source of data and information to be analysed. Further developments could include an analysis of emojis as used by other authors [40].

As part of future work, more versatile programming tools and their libraries should be explored for a deeper analysis. This includes the use of Python’s tool, namely Twython, which is specifically meant for Twitter analysis. At the same time, it integrates well with Natural Language Toolkit (NLTK), which has machine learning and Text2emotion enhanced features [41]. This would also allow us to connect the emotions associated with emojis.

## 7. Conclusions

In this paper, a methodology for a sentence-based sentiment and emotional analysis from Twitter scripts for evaluating risk perception with the Herculaneum Archaeological Park during the COVID-19 pandemic was developed. It illustrated the importance of emotional factors associated with this site.

It also highlighted the fact that the place prevailed over the circumstances, demonstrating that the Archaeological Park of Herculaneum has its own power and is able to give confidence and serenity, despite the surrounding conditions being adverse, reducing the perception of risk.

The methodology offers a valuable means for providing continuous feedback on the perceived risk of the site. In the future, this functionality could be extended to incorporate any mitigation actions and then re-evaluate the perceived risk. Such feedback will help restore more confidence to visitors.

## Figures and Tables

**Table 1:** Keywords used search terms.

Keyords—Italian/English	Kewords—English Translation
Ercolano parco	Herculaneum park
Ercolano scavi	Herculaneum excavations
Herculaneum ruins	-
Herculaneum site	-

**Table 2:** Phases and descriptions during COVID-19 pandemic in Italy.

Phase	Period	Description
1	From 30 June 2020 to 30 September 2020.	Partial normality was re-established except for limited entry in certain locations and the use of face masks in closed spaces.
2	From 1 October 2020 to 31 October 2020.	Worsening of the epidemic. The closures started again, but only in certain zones.
3	From 1 November 2020 to 16 November 2020 (end of data collecting period).	Catering activities, sports centres and more general aggregation centres were closed. Later, during this phase, Italy was in semi-lockdown.

**Table 3:** Partition by NodeXL of English words used for analysis of the three phases.

Words	Count Phase 1	Count Phase 2	Count Phase 3
Words in Sentiment List#1: Positive	12	13	0
Words in Sentiment List#2: Negative	3	3	0
Words in Sentiment List#3: COVID-19	0	0	0
Non-categorized Words	284	290	176
Total Words	299	306	176

**Table 4:** Partition by NodeXL of Italian words used for analysis of the three phases.

Words	Count Phase 1	Count Phase 2	Count Phase 3
Words in Sentiment List#1: Positive	289	55	2
Words in Sentiment List#2: Negative	33	6	0
Words in Sentiment List#3: COVID-19	0	0	0
Non-categorized Words	1578	297	37
Total Words	1900	358	39

## Data Availability

Data were downloaded from Twitter in the periods indicated in the paper.
